# Isolation of Zika Virus from Febrile Patient, Indonesia

**DOI:** 10.3201/eid2205.151915

**Published:** 2016-05

**Authors:** Aditya Perkasa, Frilasita Yudhaputri, Sotianingsih Haryanto, Rahma F. Hayati, Chairin Nisa Ma’roef, Ungke Antonjaya, Benediktus Yohan, Khin Saw Aye Myint, Jeremy P. Ledermann, Ronald Rosenberg, Ann M. Powers, R. Tedjo Sasmono

**Affiliations:** Eijkman Institute for Molecular Biology, Jakarta, Indonesia (A. Perkasa, F. Yudhaputri, R.F. Hayati, C.N. Ma’roef, B. Yohan, K.S.A. Myint, R.T. Sasmono);; Siloam Hospitals Jambi, Jambi, Indonesia (S. Haryanto);; Eijkman-Oxford Clinical Research Unit, Jakarta (U. Antonjaya);; Centers for Disease Control and Prevention, Fort Collins, Colorado, USA (J.P. Ledermann, R. Rosenberg, A.M. Powers)

**Keywords:** Zika virus, Indonesia, arbovirus, viruses, vector-borne infections

**To the Editor:** Arthropodborne viruses (arboviruses) cause substantial human disease worldwide and have a pronounced effect on public health throughout Asia. Zika virus, discovered in Uganda in 1947 (*1*), is a flavivirus related to the following viruses: dengue (DENV), West Nile, Japanese encephalitis, and yellow fever. Like DENV, Zika virus is transmitted by *Aedes* mosquitoes. Zika virus emerged as a public health problem in 2007, when it caused an epidemic in Micronesia (*2*). Since then, the virus has caused epidemics elsewhere in the Pacific islands (*3*) and recently emerged in South America (*4*). Zika virus has been reported to cause mild and self-limited infection that can be misdiagnosed as dengue because of similar clinical features and serologic cross-reactivity (*2*). Zika virus has not, however, been reported to cause substantial thrombocytopenia or result in the serious vascular leakage that can be fatal in DENV infection.

Until recently, most evidence for Zika virus infection in Asia, including in Indonesia (*5*), has been serologic, but recent virus strains isolated from persons in Thailand (*6*), the Philippines (*7*), and Cambodia (*8*) have begun to clarify its genomic diversity. Phylogenetically, Zika virus appears to have 2 major lineages, African and Asian (*9*).

During December 2014–April 2015, a confirmed outbreak of dengue (determined by reverse transcription PCR [RT-PCR] for DENV and nonstructural protein 1 [NS1] antigen detection; data not shown) occurred in Jambi Province, central Sumatra, Indonesia. We received samples from 103 case-patients with clinically diagnosed dengue; these samples had been negative for DENV by RT-PCR, NS1 antigen detection, or evidence of seroconversion by ELISA (data not shown). We tested the samples for other viruses using alphavirus and flavivirus RT-PCR (targeting genome positions 6533–6999 and 8993–9258, respectively). In parallel, we attempted virus isolation using Vero cells. 

One sample, JMB-185, came from a 27-year-old man who sought treatment at the Jambi city hospital 2 days after illness onset with a sudden high fever, headache, elbow and knee arthralgia, myalgia, and malaise. He did not exhibit some common clinical characteristics of Zika virus infection (*10*), including maculopapular rash, conjunctivitis, or peripheral edema. Hematologic testing revealed lymphocytopenia and monocytosis; platelet count was within reference range. Results of all assays were negative for DENV, including NS1 antigen detection with NS1 Ag Rapid Test (SD Bioline, Kyong, South Korea); PanBio Dengue Early NS1 ELISA (Alere, Brisbane, Australia); PanBio Dengue Duo IgM and IgG ELISA (Alere); and Simplexa real-time RT-PCR (Focus Diagnostics, Cypress, CA, USA). The illness was self-limiting, and the patient recovered 2 days after he sought treatment without any complications.

Of the 103 DENV-negative specimens we tested, only JMB-185 was positive for flavivirus and displayed cytopathic effects when cultured in Vero cells for 10 days. A subsequent passage was performed, and supernatants from both passages were tested for flaviviruses by RT-PCR. A 265-bp amplicon was generated from JMB-185 by using flavivirus consensus primers. This consensus amplicon product had ≈85% nucleotide identity with the prototype Zika virus (strain MR 766, 1947, Uganda). An additional larger amplicon was generated (nt 9278–9808 of NS5 gene), and a phylogenetic tree was constructed based on the partial sequence of the NS5 region (530 bp) for JMB-185 (GenBank accession no. KU179098) and other Zika virus sequences, including those from Cambodia, Yap Island, Thailand, and the Philippines (Figure). Phylogenetic analysis indicated that JMB-185 belonged to the Zika virus Asian lineage and had 99.24% nucleotide identity to an isolate from a Canadian visitor to Thailand (*10*). It was also close to a Zika virus strain isolated from an Australian traveler who had visited Java (on the basis of a different NS5 region; data not shown). The original serum and passage samples were further tested with Zika virus–specific real-time quantitative RT-PCR (*2*) by using the QuantiTect Probe RT-PCR Kit (QIAGEN, Valencia, CA, USA) with amplification in the iCycler iQ5 (Bio-Rad, Hercules, CA, USA), following the manufacturer’s instructions. Viral titers of JMB-185, as determined by real-time quantitative RT-PCR, were 4.25 × 10^3^ PFU, 5.07 × 10^7^ PFU, and 7.33 × 10^6^ PFU for the clinical sample, first passage, and second passage, respectively.

**Figure:**
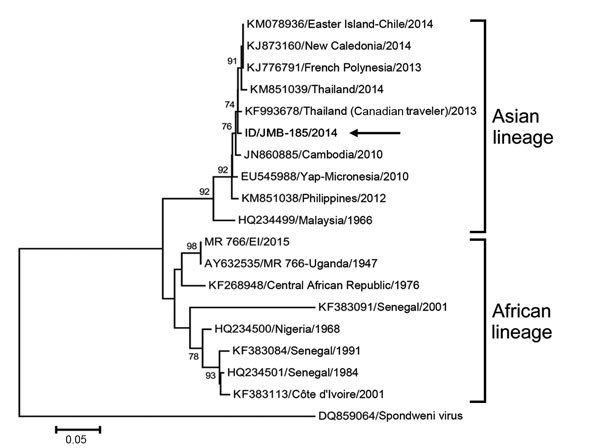
Phylogenetic tree comparing Zika virus isolate from a patient in Indonesia (ID/JMB-185/2014; arrow) to reference strains from GenBank (accession numbers indicated). The tree was constructed from nucleic acid sequences of 530 bp from the nonstructural protein 5 region by using the minimum evolution algorithm in MEGA 6 (http://www.megasoftware.net). Numbers to the left of the nodes are bootstrap percentages (2,000 replications). Bootstrap values <70 are not shown. The tree was rooted with the Spondweni virus isolated in South Africa as the outgroup. Scale bar indicates nucleotide substitutions per site.

The isolation and characterization of Zika virus from a resident with no travel history confirm that the virus is circulating in Indonesia and that, by mimicking mild dengue infection, this infection is likely contributing to the large number of undiagnosed cases of acute febrile illness. Although reported human cases of Zika virus infection have been rare in Southeast Asia (*1*), confusion with dengue and difficulty in obtaining a laboratory diagnosis are likely causing its incidence to be underestimated. Surveillance must be implemented to evaluate and monitor the distribution of Zika virus and the potential public health problems it may cause in Indonesia.

## References

[1] Dick GWA, Kitchen SF, Haddow AJ. Zika virus. I. Isolations and serological specificity. Trans R Soc Trop Med Hyg. 1952;46:509–20. 10.1016/0035-9203(52)90042-4 12995440

[2] Lanciotti RS, Kosoy OL, Laven JJ, Velez JO, Lambert AJ, Johnson AJ, Genetic and serologic properties of Zika virus associated with an epidemic, Yap State, Micronesia, 2007. Emerg Infect Dis. 2008;14:1232–9. 10.3201/eid1408.080287 18680646 PMC2600394

[3] Musso D, Nilles EJ, Cao-Lormeau VM. Rapid spread of emerging Zika virus in the Pacific area. Clin Microbiol Infect. 2014;20:O595–6. 10.1111/1469-0691.12707 24909208

[4] Zanluca C, de Melo VCA, Mosimann ALP, Dos Santos GIV, Dos Santos CND, Luz K. First report of autochthonous transmission of Zika virus in Brazil. Mem Inst Oswaldo Cruz. 2015;110:569–72. 10.1590/0074-02760150192 26061233 PMC4501423

[5] Olson JG, Ksiazek TG. Suhandiman, Triwibowo. Zika virus, a cause of fever in Central Java, Indonesia. Trans R Soc Trop Med Hyg. 1981;75:389–93. 10.1016/0035-9203(81)90100-0 6275577

[6] Buathong R, Hermann L, Thaisomboonsuk B, Rutvisuttinunt W, Klungthong C, Chinnawirotpisan P, Detection of Zika virus infection in Thailand, 2012–2014. Am J Trop Med Hyg. 2015;93:380–3. 10.4269/ajtmh.15-0022 26101272 PMC4530765

[7] Alera MT, Hermann L, Tac-An IA, Klungthong C, Rutvisuttinunt W, Manasatienkij W, Zika virus infection, Philippines, 2012. Emerg Infect Dis. 2015;21:722–4. 10.3201/eid2104.141707 25811410 PMC4378478

[8] Heang V, Yasuda CY, Sovann L, Haddow AD, Travassos da Rosa AP, Tesh RB, Zika virus infection, Cambodia, 2010. Emerg Infect Dis. 2012;18:349–51. 10.3201/eid1802.111224 22305269 PMC3310457

[9] Haddow AD, Schuh AJ, Yasuda CY, Kasper MR, Heang V, Huy R, Genetic characterization of Zika virus strains: geographic expansion of the Asian lineage. PLoS Negl Trop Dis. 2012;6:e1477. 10.1371/journal.pntd.0001477 22389730 PMC3289602

[10] Fonseca K, Meatherall B, Zarra D, Drebot M, MacDonald J, Pabbaraju K, First case of Zika virus infection in a returning Canadian traveler. Am J Trop Med Hyg. 2014;91:1035–8 . 10.4269/ajtmh.14-0151 25294619 PMC4228871

